# Vector-Borne Diseases - constant challenge for practicing veterinarians: recommendations from the CVBD World Forum

**DOI:** 10.1186/1756-3305-5-55

**Published:** 2012-03-20

**Authors:** Gad Baneth, Patrick Bourdeau, Gilles Bourdoiseau, Dwight Bowman, Edward Breitschwerdt, Gioia Capelli, Luís Cardoso, Filipe Dantas-Torres, Michael Day, Jean-Pierre Dedet, Gerhard Dobler, Lluís Ferrer, Peter Irwin, Volkhard Kempf, Babara Kohn, Michael Lappin, Susan Little, Ricardo Maggi, Guadalupe Miró, Torsten Naucke, Gaetano Oliva, Domenico Otranto, Banie Penzhorn, Martin Pfeffer, Xavier Roura, Angel Sainz, Susan Shaw, SungShik Shin, Laia Solano-Gallego, Reinhard Straubinger, Rebecca Traub, Alexander Trees, Uwe Truyen, Thierry Demonceau, Ronan Fitzgerald, Diego Gatti, Joe Hostetler, Bruce Kilmer, Klemens Krieger, Norbert Mencke, Cláudio Mendão, Lourdes Mottier, Stefan Pachnicke, Bob Rees, Susanne Siebert, Dorothee Stanneck, Montserrat Tarancón Mingote, Cristiano von Simson, Sarah Weston

**Affiliations:** 1 Koret School of Veterinary Medicine, The Robert H. Smith Faculty of Agriculture, Food and Environment, The Hebrew University of Jerusalem, Rehovot, Israel; 2 Unité de Dermatologie, Parasitologie CE, Mycologie, Ecole Nationale Vétérinaire, Agroalimentaire et de l'Alimentation, Nantes-Atlantique (ONIRIS), France; 3 Unité de Parasitologie, Mycologie, Maladies Parasitaires, Ecole Nationale Vétérinaire de Lyon, Marcy L'Etoile, France; 4 Department Microbiology & Immunology, College of Veterinary Medicine, Cornell University, Ithica, NY, USA; 5 Department of Clinical Sciences, College of Veterinary Medicine, North Carolina State University, Raleigh, NC, USA; 6 Laboratorio di Parassitologia e Ecopatologia, Istituto Zooprofi lattico Sperimentale delle Venezie, Legnaro-Padova, Italy; 7 Department of Veterinary Sciences, University of Trás-os-Montes e Alto Douro, Vila Real, Portugal; 8 Centro de Pesquisas Aggeu Magalhães, Fundação Oswaldo Cruz, Recife, Brazil; 9 School of Veterinary Sciences, University of Bristol, Langford, Bristol, UK; 10 Laboratoire de Parasitologie-Mycologie du CHU de Montpellier, UMR MIVEGEC, Centre National de Référence des Leishmania, Montpellier, France; 11 Department of Virology and Rickettsiology, Bundeswehr Institute of Microbiology, Munich, Germany; 12 Department of Animal Medicine and Surgery, Universitat Autònoma de Barcelona, Bellaterra, Barcelona, Spain; 13 School of Veterinary and Biomedical Sciences, Division of Health Sciences, Murdoch University, Murdoch, Western Australia, Australia; 14 Institut für Medizinische Mikrobiologie und Krankenhaushygiene, Klinikum der Goethe-Universität, Frankfurt am Main, Germany; 15 Clinic of Small Animals, Faculty of Veterinary Medicine, Free University of Berlin, Berlin, Germany; 16 Department of Clinical Sciences, College of Veterinary Medicine and Biomedical Sciences, Colorado State University, Fort Collins, CO, USA; 17 Department of Pathobiology, Center for Veterinary Health Sciences, Oklahoma State University, Stillwater, OK, USA; 18 Intracellular Pathogens Research Laboratory, College of Veterinary Medicine, North Carolina State University, Raleigh, NC, USA; 19 Dpto. Sanidad Animal, Facultad de Veterinaria, Universidad Complutense de Madrid, Madrid, Spain; 20 Department of Zoology - Division of Parasitology, University of Hohenheim, Stuttgart, Germany; 21 Dip. to di Scienze Cliniche Veterinarie, Universita di Napoli, Naples, Federico II, Italy; 22 Department of Veterinary Public Health, Faculty of Veterinary Medicine, University of Bari, Valenzano, Bari, Italy; 23 Department of Veterinary Tropical Diseases, Faculty of Veterinary Science, University of Pretoria, Onderstepoort, Pretoria, South Africa; 24 Institut für Tierhygiene und Öffentliches Veterinärwesen, Veterinärmedizinische Fakultät, Universität Leipzig, Leipzig, Germany; 25 Servei de Medicina Interna, Hospital Clínic Veterinari, Facultat de Veterinària, Universitat Autònoma de Barcelona, Bellaterra (Barcelona), Spain; 26 Dpto. de Medicina y Cirugía Animal, Facultad de Veterinaria, Universidad Complutense de Madrid, Madrid, Spain; 27 School of Clinical Veterinary Science, University of Bristol, Langford, Bristol, UK; 28 College of Veterinary Medicine, Chonnam National University, Gwangju, South Korea; 29 Department of Animal Medicine and Surgery, Universitat Autònoma de Barcelona, Bellatorra, Barcelona, Spain; 30 Lehrstuhl für Bakteriologie und Mykologie, Veterinärwissenschaftliches Department, Tierärztliche Fakultät, Ludwig-Maximilians-Universität München, Munich, Germany; 31 School of Veterinary Science, The University of Queensland, Queensland, Australia; 32 Infection Biology Group, Comparative Molecular Medicine, School of Veterinary Science, University of Liverpool, Liverpool, UK; 33 Institut für Tierhygiene und Öffentliches Veterinärwesen, Veterinärmedizinische Fakultät, Universität Leipzig, Leipzig, Germany; 34 Bayer Santé SAS, Puteaux, France; 35 Bayer Plc, Animal Health Division, Newbury, UK; 36 Bayer HealthCare Italy, Animal Health Division, Milan, Italy; 37 Bayer HealthCare LLC, Animal Health, Shawnee Mission, KS, USA; 38 Bayer Inc., Bayer HealthCare - Animal Health, Toronto, Canada; 39 Bayer Animal Health GmbH, Monheim, Germany; 40 Bayer Animal Health GmbH, Monheim, Germany; 41 Bayer Portugal S.A., Animal Health Division, Carnaxide, Portugal; 42 Bayer Animal Health GmbH, Monheim, Germany; 43 Bayer Vital GmbH, Leverkusen, Germany; 44 Bayer Australia, Animal Health Division, Brisbane, Australia; 45 Bayer Animal Health GmbH, Monheim, Germany; 46 Bayer Animal Health GmbH, Monheim, Germany; 47 Bayer HealthCare Spain, Animal Health Division, Barcelona, Spain; 48 Bayer HealthCare LLC, Animal Health, Shawnee Mission, KS, USA; 49 Bayer New Zealand Ltd, Auckland, New Zealand

## Abstract

The human-animal bond has been a fundamental feature of mankind's history for millennia. The first, and strongest of these, man's relationship with the dog, is believed to pre-date even agriculture, going back as far as 30,000 years. It remains at least as powerful today. Fed by the changing nature of the interactions between people and their dogs worldwide and the increasing tendency towards close domesticity, the health of dogs has never played a more important role in family life. Thanks to developments in scientific understanding and diagnostic techniques, as well as changing priorities of pet owners, veterinarians are now able, and indeed expected, to play a fundamental role in the prevention and treatment of canine disease, including canine vector-borne diseases (CVBDs).

The CVBDs represent a varied and complex group of diseases, including anaplasmosis, babesiosis, bartonellosis, borreliosis, dirofilariosis, ehrlichiosis, leishmaniosis, rickettsiosis and thelaziosis, with new syndromes being uncovered every year. Many of these diseases can cause serious, even life-threatening clinical conditions in dogs, with a number having zoonotic potential, affecting the human population.

Today, CVBDs pose a growing global threat as they continue their spread far from their traditional geographical and temporal restraints as a result of changes in both climatic conditions and pet dog travel patterns, exposing new populations to previously unknown infectious agents and posing unprecedented challenges to veterinarians.

In response to this growing threat, the CVBD World Forum, a multidisciplinary group of experts in CVBDs from around the world which meets on an annual basis, gathered in Nice (France) in 2011 to share the latest research on CVBDs and discuss the best approaches to managing these diseases around the world.

As a result of these discussions, we, the members of the CVBD Forum have developed the following recommendations to veterinarians for the management of CVBDs.

## Recommendations

1. **Forget about 'exotic disease': **Veterinarians should understand that the concept of 'exotic' diseases is no longer applicable - any disease can appear in your practice, wherever you are based, and it is your responsibility to maintain a working knowledge of these diseases, current local and regional vector trends and threats associated with client travel destinations, in order to ensure accurate diagnosis.

2. **Stay abreast of latest research: **Research efforts in the field are continually discovering new CVBDs, and new information about existing CVBDs. Again, it is the duty of the veterinarian to remain abreast of these developments.

3. **Prevention is the best approach: **Prevention of transmission is the best method of management for CVBDs. Fortunately, we know that there are a limited number of vectors involved - ticks, fleas, sand flies and mosquitoes - and preventative parasiticides are available either by chemoprophylaxis against the agent itself (as in dirofilariosis) or against the ectoparasite vectors. Veterinarians should be aware that pathogen transmission can occur almost immediately from the bites of fleas, sand flies and in some cases from ticks. Therefore, repellency is an important concept as preventative strategy to avoid a blood meal by the arthropod vectors. As a result of the changes in distribution of vectors, along with increasing frequency of pet travel, it is also vital to look to protecting pets from as many vectors as possible. This applies even in areas where some of these vectors are not traditionally endemic. Furthermore, changes in lifestyle and climate also mean that it is advisable to ensure that protection, and tick and flea control in particular, is maintained year round.

4. **Fleas are more than just a nuisance: **Fleas have been found to be vectors of a number of CVBDs and tapeworm, but are often overlooked risk factors compared with ticks, sand flies and mosquitoes. Flea prevention is just as important as protection from other vectors from both a veterinary and public health perspective.

5. **Sand flies are the only proven vector of leishmaniosis, but in non-endemic areas vets should be aware of non-vectorial routes of transmission: **While sand flies are the only proven vector for leishmaniosis, in rare cases the organism could potentially also be transmitted by other routes (as *e.g*. vertical or venereal transmission, or blood transfusion). As a result, especially in non-endemic areas, leishmaniosis should still be considered as a potential diagnosis if there is a possibility of non-vectorial transmission from an exposed dog.

6. **Know the travel schedule of your clients: **While any dog can be affected, particular attention should be paid to the health of dogs that travel or are imported from other regions or are in contact with travelling dogs. These dogs are at risk themselves, but also potentially pose a threat to the health of local animals.

7. **As diagnosis can be complex and challenging, consider all options: **Diagnosis of CVBDs is complex as there are often no specific clinical signs and clinic-pathological abnormalities, varied presentations and the presence in many cases of asymptomatic infection, posing a hidden risk to the dog of future emergent diseases. It is also important to consider possible co-infections, as these are common, and can lead to complex manifestations and challenging diagnoses. When selecting diagnostic procedures, alongside ensuring the use of scientifically agreed standards, it may be necessary to combine multiple tests in order to rule out infection/co-infections.

8. **Understand that treatment may not be the end of the story: **Once diagnosed, treatment of CVBDs is often challenging, in part due to the lack of effective treatments, and the difficulty in achieving full elimination of the pathogen from the animal, even when achieving a 'clinical cure'. As a result, treated dogs can remain as 'Trojan horse' reservoirs of disease for dogs or other animals in the vicinity, and should therefore be treated with preventative compounds to minimise the risk of pathogen transmission by the competent vectors.

9. **Engage with your clients to improve outcomes: **Routine, regular client education through delivery of the annual health check is an essential aspect of veterinary practice, and ensuring clients understand their role in managing routine parasiticide use, vector exposure and reporting signs is critical to an effective CVBD management programme.

10. **Alert public health authorities where appropriate: **Veterinarians are at the forefront of efforts to manage the risk of zoonotic disease. When treating animals with CVBDs, remember that they may act as sentinels for disease in the human population, and where appropriate, ensure local health authorities are notified. Veterinarians have a vital role to play in the 'One Health' approach to prevention of infectious disease.

In conclusion, we believe that while CVBDs are a growing threat to the health of dogs worldwide, it is within the power of veterinarians and pet owners to manage this threat through the use of effective preventative treatments, education and awareness. We call on all veterinarians wherever they are based to implement rigorous CVBD management programmes based on the principles outlined above to make it a global and thus effective strategy to reduce the burden of CVBDs.

## Competing interests

The authors declare that they have no competing interests. The authors are members of the CVBD World Forum. The CVBD World Forum was founded during the 1st International CVBD Symposium in April 2006 in Billesley, UK, as a consequence of the increasing global threats through vector-borne diseases. The CVBD World Forum is supported by Bayer Animal Health.

## Authors' contributions

All authors contributed equally to this work. All authors read and approved the final manuscript. 

